# Super-Selective Trans-Catheter Arterial Embolization (TAE) of the Vesical Arteries in the Management of Intractable Hematuria Secondary to Advanced Bladder and Prostate Cancers

**DOI:** 10.7759/cureus.58016

**Published:** 2024-04-11

**Authors:** Ameer Alarayedh, Sharif Abdulwahab, Mohamed Mubarak

**Affiliations:** 1 Urology, Salmaniya Medical Complex, Manama, BHR; 2 Radiology, Salmaniya Medical Complex, Manama, BHR

**Keywords:** palliative care, super-selective tae, vesical artery embolization, advanced bladder cancer, transarterial embolization (tae), locally advanced prostate cancer, bladder cancer, refractory hematuria

## Abstract

This article was previously presented as an abstract at the 18th UAA Congress, Seoul, October 15-17, 2020, and the abstract was published in The International Journal of Urology. It was also presented as an e-poster at the 2021 BAUS Annual Meeting on June 22, 2021.

Introduction

In frail patients intractable hematuria secondary to advanced pelvic malignancies is a clinical challenge. Super-selective TAE of the vesical arteries is a suitable minimally invasive option. We present our experience in this patient cohort.

Patients and methods

All patients who underwent TAE from January 2014 to December 2019 were included. Super-selective TAE of the superior and inferior vesical arteries was done using 300-500µ polyvinyl alcohol (PVA) particles. Demographic data, cancer stage, associated urinary system obstruction, pre-embolization palliative treatment, chemotherapy, and radiotherapy were recorded. Technical and clinical success, time to cessation of hematuria, recurrence of hematuria, and complications were recorded. Data are presented as mean ± standard deviation, and statistical significance is set at p<0.05.

Results

From 2014 to 2019, seven patients underwent eight procedures. The average patient’s age was 60.6±10.3 years. All presented with gross hematuria, six due to locally advanced and/or metastatic bladder cancer, and one due to prostate cancer. The average time of hematuria clearance was 60 hours. The average hemoglobin levels at the time of the procedure, one month, and six months post-embolization were 9.6±1.7 g/dL, 10.6±1.5 g/dL (p<0.05), and 9.6±0.9 g/dL, respectively (p>0.05). Packed red blood cell (PRBC) requirements decreased from 7±2 units to 5±3 units after the procedure (p >0.05). The patients were followed up for an average of 13.6 months and four had a recurrence at an average of four months post-embolization.

Conclusion

Super-selective TAE is an effective palliative method in controlling intractable hematuria. The risks of major surgery and anesthesia are omitted, and the procedure can be repeated as needed. Furthermore, post-embolization complications, using this technique, are minor and manageable.

## Introduction

Managing intractable hematuria associated with advanced bladder and prostate malignancies poses a formidable clinical challenge, particularly in patients deemed unsuitable for general anesthesia. While the initial approach may appear straightforward, complexities emerge when we consider the patient's preferences and carefully assess the risk-to-benefit ratios associated with various treatment options. The first line of treatment is supportive, mainly via urethral catheterization, bedside bladder washout, and continuous bladder irrigation [1,2]. If supportive therapy fails, cystoscopy with clot evacuation and fulguration may be performed [1,2]. Failure of these methods creates a genuine therapeutic dilemma for the urologist, especially when radical approaches are unsuitable for frail or terminally ill patients. For instance, radical cystectomy and/or open bilateral ligation of the internal iliac arteries, while effective, carry substantial risks, especially in elderly, comorbid, or terminally ill patients [3]. Therefore, the selection of the most appropriate procedure demands meticulous consideration of the potential benefits weighed against the associated risks [3].

The introduction and development of the trans-catheter arterial embolization (TAE) of the internal iliac artery and its branches have provided a minimally invasive alternative to direct surgical intervention aiming to control intractable hematuria secondary to advanced pelvic malignancies in this patient cohort [4]. Although TAE of the vesical artery was first described many decades ago, only a limited number of patients are reported in the literature. In this study, we aim to report our experience with TAE using super-selective techniques to manage intractable hematuria from advanced pelvic urological malignancies.

## Materials and methods

This is a retrospective case series of all patients who had inoperable or recurrent malignant neoplasms of the bladder and prostate with loco-regional infiltration. All patients who presented with intractable hematuria and subsequently had TAE were included. Intractable was defined as the failure of conservative treatment. The study was conducted at Salmaniya Medical Complex, Kingdom of Bahrain, from January 2014 to December 2019 and was approved by our institutional ethics committee.

Before the procedure, the patients were worked up with a complete blood count, coagulation profile, and basic metabolic profile. All patients were on a three-way Foley catheter. TAE was performed by a single interventional radiologist using a single-plane dedicated angiography machine (Siemens Artist Zee). A single puncture with a 5 Ch sheath and selective catheterization of the internal iliac artery on both sides using a 4-5 Ch cobra catheter with the subsequent super-selective catheterization of the superior vesical artery using a 2.7 Ch micro-catheter (Prograte, Terumo) were done. Embolization was done with 300-500 µ polyvinyl alcohol (PVA) particles (Cook Medical, Merit Medical), and a maximum of two vials were used for both sides. The endpoint for embolization was the anatomical cessation of blood flow to the bladder wall or the tumor's vascular bed. Manual femoral puncture hemostasis was performed with no closure device. All procedures were done without sedation, and patients were discharged from the angiography suite to the ward with postoperative instructions.

Demographic and clinical data were collected from the patient's medical records, nursing notes, and daily observation charts. Data collected includes gender, age at presentation, type of malignancy, histological grade, clinical stage, previous chemoradiation therapy, hematuria control, response to TAE, and post-procedural complications. The patients' characteristics, clinical presentation, intervention efficiency, and outcomes are reported. Clinical success was defined as the cessation of the hematuria within 30 days of the procedure. Short- and long-term effectiveness and mortality were recorded. Radiographic findings and the technical success of TAE procedures were analyzed, and the findings are presented. Analyses were done using the Statistical Package for Social Sciences (SPSS version 23). Data are expressed as mean ± standard deviation. Patient data were compared and analyzed using a student's t-test, ANOVA, and chi-squared test where applicable. All reported statistically significant p-values were <0.05.

## Results

Over five years, we had seven patients (six males and one female) who underwent eight angioembolization procedures. The patients' average age at the time of the procedure was 60.6±10.3 years (range: 51-77 years). TAE was performed due to intractable hematuria secondary to bladder cancer (87.5%) and prostate cancer (12.5%). There was upper urinary tract involvement in three patients (42.8%). Three patients had metastatic bladder cancer (42.8%), two had locally advanced bladder cancer (28.5%), and one had metastatic prostate cancer (14.3%). The initial pathological tumor types were high-grade papillary urothelial carcinomas of the bladder and one moderately differentiated adenocarcinoma of the prostate with bladder base invasion. One patient with locally advanced bladder cancer treated with trimodal therapy had cystoscopic evidence of radiation-related cystitis, which was the cause of bleeding (Table 1).

**Table 1:** Patient characteristics

Patient	Age	Gender	Presenting complaint	Etiology	Urinary system involvement	TNM	Chemotherapy	Radiotherapy
1	54	M	Hematuria	Urothelial cancer	Bladder	T3bN3M0	No	No
2	54	M	Hematuria	Urothelial cancer	Bladder with renal and ureteric involvement	T4N3M1	No	No
3	73	M	Hematuria	Prostate adenocarcinoma	Prostate with bladder involvement	T4N0M1c	Docetaxel	Yes
4	54	F	Hematuria	Urothelial cancer	Bladder with renal and ureteric involvement	T3N2M1	Carboplatin gemcitabine	Yes
5	51	M	Hematuria	Urothelial cancer	Bladder with renal and ureteric involvement	T4N3M1	No	Yes
6	77	M	Hematuria	Urothelial cancer	Bladder	T4aN0M0	Cisplatin	Yes
7	68	M	Hematuria	Urothelial cancer	Bladder	T3bN0M0	No	No

**Figure 1:**
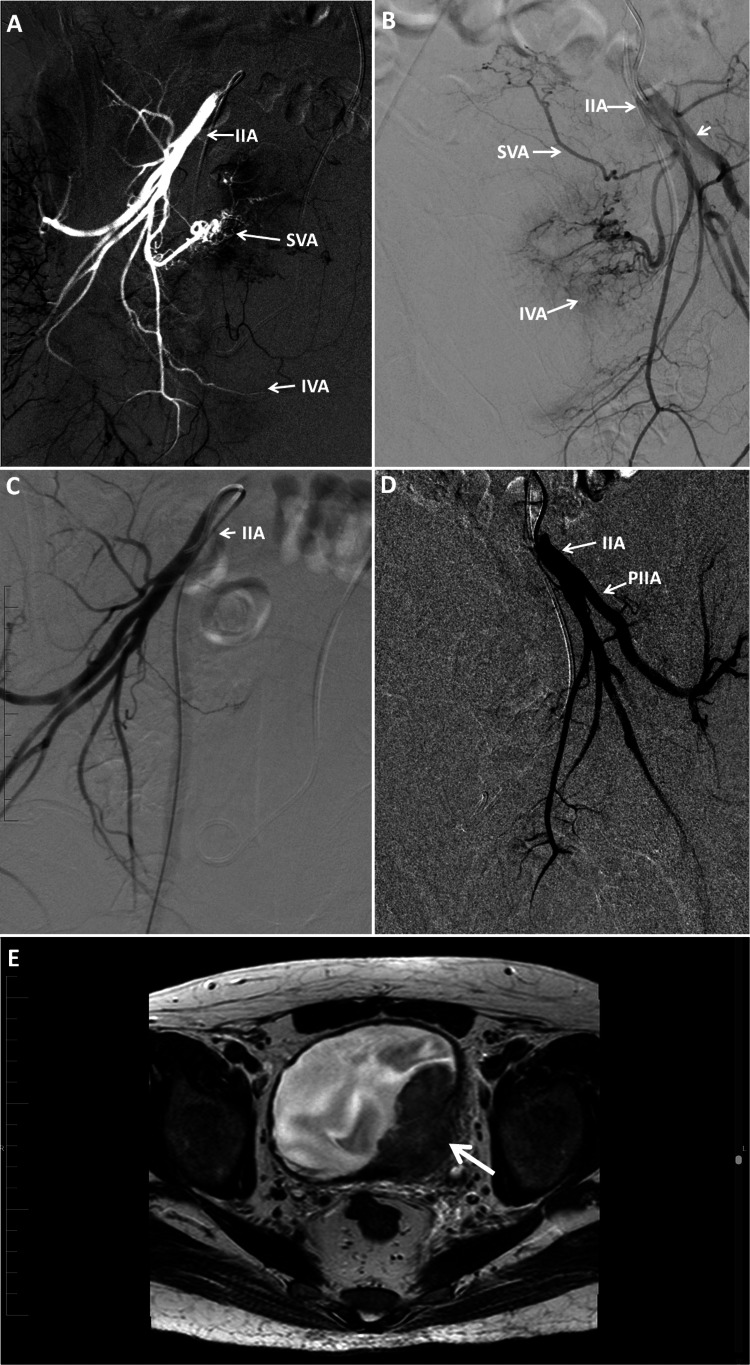
Patient 1: (A) right angiogram showing hypervascularity. (B) Left angiogram showing hypervascularity. (C) Right angiogram post-embolization showing a reduction in blood flow to the vesical arteries. (D) Left angiogram post-embolization showing cessation of flow to the vesical arteries. (E) MRI with a large tumor involving the posterolateral bladder wall with peri-vesical fat invasion. IIA, internal iliac artery; PIIA, posterior branch of internal iliac artery; SVA, superior vesical artery; IVA, inferior vesical artery; MRI, magnetic resonance imaging

**Figure 2:**
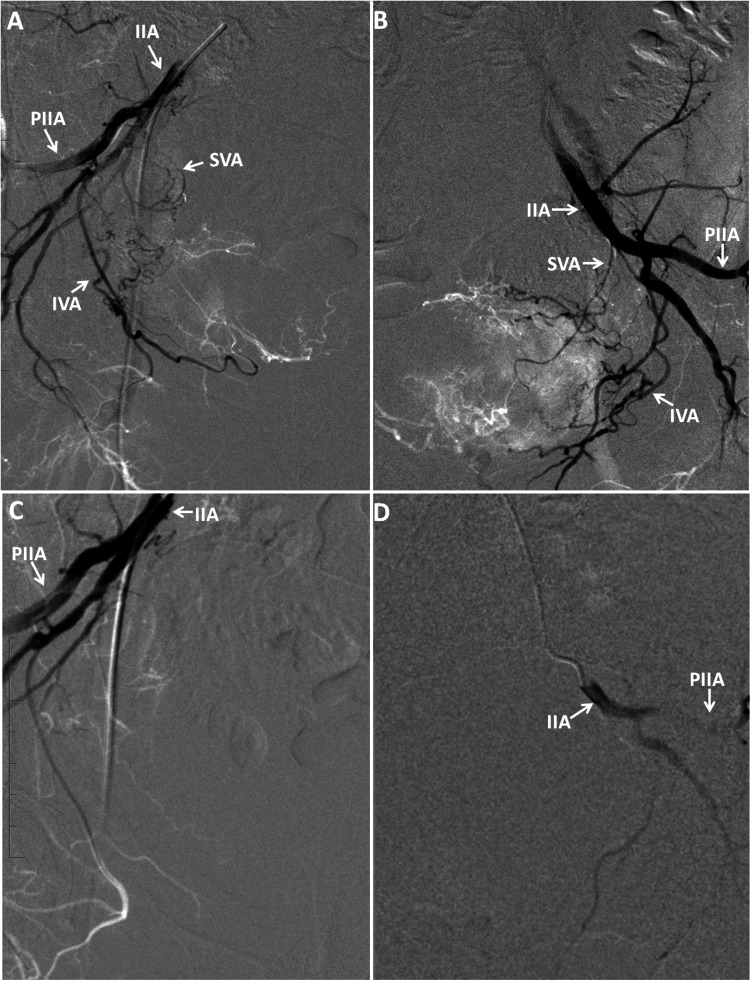
Patient 2: (A) right angiogram showing hypervascularity. (B) Left angiogram showing hypervascularity. (C) Right angiogram post-embolization showing complete thrombosis of the vesical arteries. (D) Left angiogram post-embolization showing complete thrombosis of the vesical arteries. IIA, internal iliac artery; PIIA, posterior branch of internal iliac artery; SVA, superior vesical artery; IVA, inferior vesical artery

**Figure 3:**
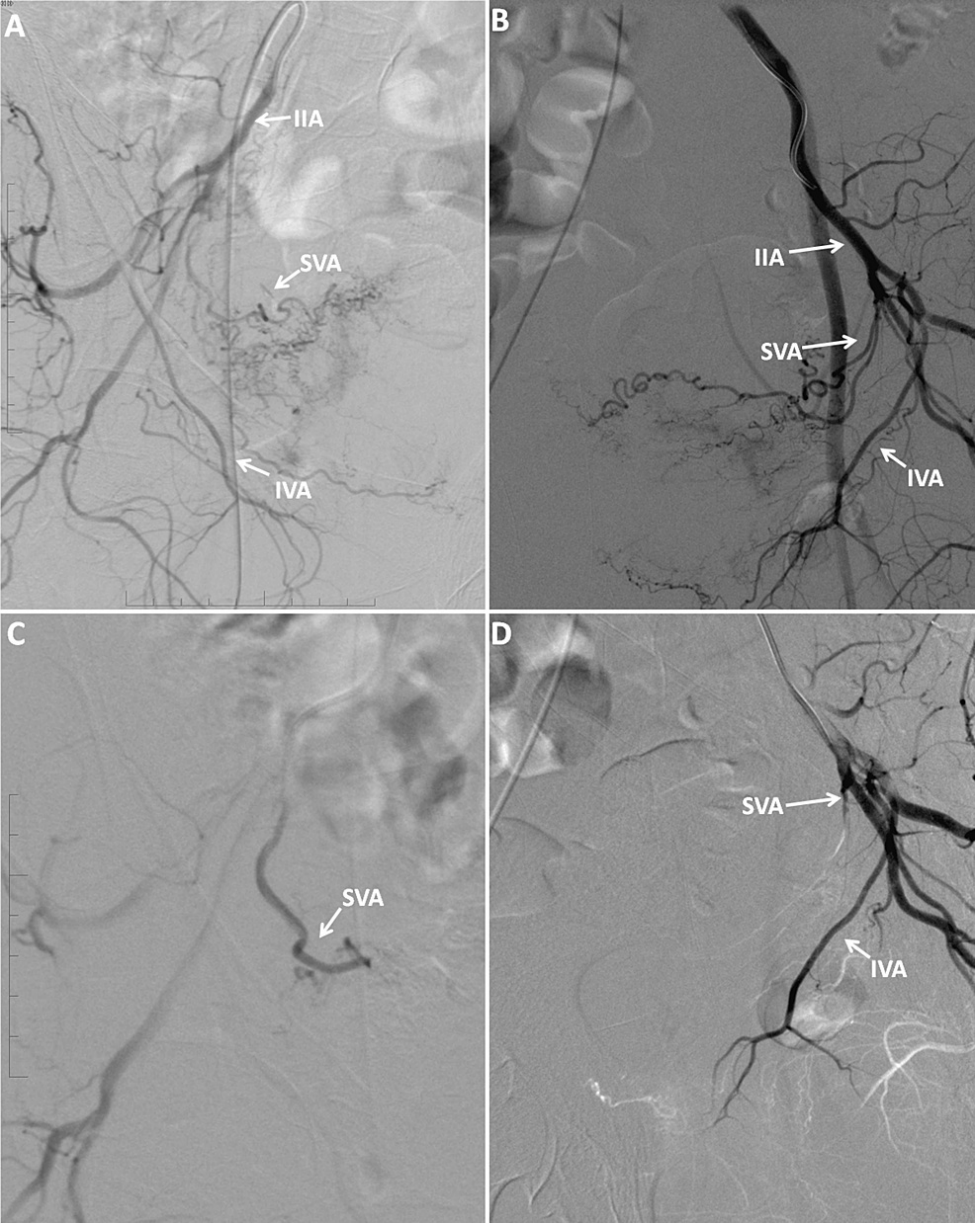
Patient 4: (A) Right angiogram showing hypervascularity. (B) Left angiogram showing hypervascularity. (C) Right angiogram post-embolization of SVA only, showing cessation of flow. (D) Left angiogram post-embolization of SVA only. IIA, internal iliac artery; PIIA, posterior branch of internal iliac artery; SVA, superior vesical artery; IVA, inferior vesical artery

All of the patients presented with frank hematuria. Bladder irrigation and endoscopic means to control the hematuria were used in six patients but all were unsuccessful. Cystectomy was considered in four patients; however, only one patient had a cystectomy done in an external center two years after embolization. The average time from the onset of hematuria until the procedure was 29.8±16.6 days (range: 0-56 days). One patient, who was already on dual antiplatelet therapy, had a massive hemorrhage during an attempt to control the bleeding endoscopically and immediately underwent the TAE procedure. The majority of the patients showed mild hypervascularity in the bladder mucosa with no active bleeding at the time of examination (Figures 1-3).

PVA particles 300-500 microns were used as an embolic agent. Technical success and clinical success were achieved in 100% and 87.5% of the cases, respectively. The average time of hematuria clearance was 60±64.1 hours (range: 0-168 hours). All had bilateral superior and/or inferior vesical artery embolization except one patient who underwent unilateral embolization with an early recurrence of the hematuria. Hemoglobin levels before and after TAE were 9.6±1.7 g/dL and 9.4±1.7 g/dL, respectively. Furthermore, on one and six months of follow-up, hemoglobin levels were 10.6±1.5 g/dL (p<0.05) and 9.6±0.9 g/dL, respectively. The TAE effect on hematocrit level changes was statistically insignificant with average levels of 31.3±2.7 before and 30.1±4 after the procedures. However, following the procedures, the average packed red blood cell (PRBC) transfusion requirement decreased from 7.3±2 to 5±3.3 units. 

The most common post-embolization complications were abdominal/suprapubic pain, urgency, frequency, and dysuria. Follow-up was carried out for all patients and lasted between two months to three years with an average of 13.6±14 months. Less than half of the cases done had a recurrence of hematuria at approximately four months post-procedure (range 0.3-7 months) (Table 2)

**Table 2:** Pre- and post-embolization parameters

Patient	Presentation to embolization	Hematuria clearance	Hematuria recurrence	Site of embolization	Embolization laterality	Vascularity	Technical success	Clinical success	Complications	Follow-up (months)
1	3 weeks	0 hours	7 months	Superior & inferior vesical A.	Bilateral	Hypervascularity	Yes	Yes	Suprapubic and penile pain	7
2	8 weeks	72 hours	N/A	Inferior vesical A.	Bilateral	Hypervascularity	Yes	Yes	None	2
3	2 weeks	24 hours	N/A	Superior & inferior vesical A.	Bilateral	Right-hypervascularity; left-minimal increased vascularity	Yes	Yes	Frequency and urgency	6
4a	3 weeks	0 hours	6 months	Superior vesical A.	Bilateral	Mild hypervascularity	Yes	Yes	None	36 (3 years)
4b	6 weeks	1 week	N/A	Inferior vesical A.	Bilateral	Mild hypervascularity	Yes	Yes	Frequency and urgency	
5	6 weeks	96 hours	10 days	Superior & inferior vesical A.	Unilateral, right	Mild hypervascularity	Yes	No	Suprapubic pain, dysuria, frequency, and urgency	2
6	5 week	0 hours	3 months	Superior vesical A.	Bilateral	Mild hyperemia of bladder wall	Yes	Yes	Frequency and urgency	8
7	0 days	120 hours	N/A	Superior & inferior vesical A.	Bilateral	Mild hypervascularity	Yes	Yes	None	12

Currently, only one patient out of the seven described in the series is alive; all the others passed away due to disease progression.

## Discussion

Intractable hematuria secondary to advanced pelvic malignancies is a challenge that many urologists must manage [2]. This cohort usually includes elderly and frail patients with multiple comorbidities and poor general conditions [3]. Likewise in our series, most of the patients were unfit for radical surgery and the managing urologists advised against surgery. In such patients when conservative measures fail to control the hematuria, trans-catheter arterial embolization (TAE) of the vesical arteries is a good option and can yield results comparable to that of open ligation of the internal iliac artery [3,5]. Hald and Mygind initially described TAE in 1974. Further technical and instrumental developments took place to introduce different catheters, techniques, and embolic materials [4]. Refined results were achievable with the selective embolization of the anterior division of the internal iliac artery and super-selective embolization of the vesical arteries [5-7]. The latter technique was described by Kobayashi et al. in 1980 [7].

TAE appears to be commonly used in clinical practice; however, most of the literature is in the form of case reports and case series. We currently lack long-term prospective randomized controlled trials that report high-quality data regarding the efficiency, safety, and outcomes of TAE [1]. Nonetheless, in our subset of patients we have had a positive experience with TAE, and believe that it can be a very promising form of palliation. In our series, the average age of patients offered TAE was 60.6±10.3 years (range: 51-77 years). Similar age groups were seen in other reports and series. Taha et al. reviewed 38 studies encompassing 295 patients with an age range between 51 and 95 years [8]. Furthermore, many other case studies and series describe patients within a similar age range. It is, however, essential to mention that only 17.6% (n=52) of the patients reviewed by Taha et al. underwent super-selective TAE, whereas 100% of our cohort had the mentioned procedure. In our series, technical success was achieved at 100%, while clinical success was achieved at 87.5%. TAE's success rate is usually high, and multiple studies in the literature report success rates ranging from 43% to 100% [2,9,10].

A few studies went on a further step and compared their patients' peri-operative hemoglobin, hematocrit, and PRBC requirements. In El-Assmy et al., seven patients underwent TAE, and the PRBC requirement was 4.5 units (range: 2-8 units) of blood pre-operatively [11]. Halpenny et al. reported that the PRBC transfusion requirement dropped from 8.6 units to 0.3 units after embolization [12]. Furthermore, Liguori et al. stated that 55% of the patients in their study required an average of four PRBC units (range: 1-17 units), and only 30% required more PRBC after the procedure; the mean peri-operative hematocrit (27% vs 31%) and hemoglobin (8.7g/dL vs 10.3g/dL) were significantly different (p<0.01) [13]. Another study by Korkmaz et al. described similar significant peri-operative improvements in hematocrit and hemoglobin levels from 26.95% to 30.11% (p<0.01) and from 8.95 to 10.25 g/dL (p<0.01), respectively [14]. In our series transfusion requirements were higher reflecting more severe bleeding, six patients (86%) required PRBC transfusion during admission with an average of 7.3±2 and 5±3.3 PRBC units before and after TAE, respectively. We found that there was a significant difference in hemoglobin levels one-month post-embolization; nonetheless, this improvement was not sustained at six months. 

Common complications following TAE include gluteal claudication, Brown Sequard’s syndrome, bladder necrosis, skin necrosis, and gluteal paresis [8]. The most commonly reported complication is post-embolization syndrome, a clinical entity that includes fever, nausea, vomiting, and gluteal pain due to tissue necrosis [2,15]. In our series, four out of seven patients had minor postoperative complications. Four patients (57%) complained of lower urinary tract symptoms (LUTS) after the procedure, while two patients (29%) complained of suprapubic pain. Lower complication rates than the less selective approaches could probably be explained by our choice of super-selective embolization technique.

As for recurrent hematuria, early recurrence occurred in one patient ten days after the procedure. We believe that the reason for early recurrence was that only unilateral super-selective embolization was done. Late recurrence occurred in three patients (42.8%) after an average of five months from the first procedure, one of which was started on dual antiplatelet and anticoagulant therapy post-procedure. Our recurrence rate is similar to that reported in the literature. El-Assmy et al. carried out selective angioembolization in seven patients, of which three (42.8%) had recurrent hematuria within a shorter duration than our cohort [11]. Another study that exclusively carried out super-selective TAE reported that 19 out of 44 patients (43%) achieved complete hematuria cessation, whereas a second TAE was required in five [12].

Although follow-up in patients who present with intractable hematuria secondary to advanced pelvic malignancies is usually of short duration and mortality rates are high, we managed to follow-up our patients for an average of 13.6 months (range: 2-36 months). The one-month mortality rate in our case series was 0%. Similar findings were reported by Kormkaz et al. [14]. However, at six and 12 months, the mortality rate increased to 42% and 71.4%. The latter finding goes with results described by other case series that followed up with their patients for a longer duration [8,12].

## Conclusions

Our study demonstrates that bilateral super-selective TAE of the vesical arteries with PVA particles effectively stops intractable hematuria in high-risk, surgically unfit patients with advanced bladder and prostate cancers. The procedure, with minor manageable side effects, offers sustainable results and can be repeated as needed. We recommend a prospective randomized controlled study to compare TAE with expectant management for further validation.
